# Vulnerabilities in Mental Health due to Covid-19 Pandemic: The Response of the Italian Physicians

**DOI:** 10.1007/s11205-023-03111-y

**Published:** 2023-05-30

**Authors:** Alessandra De Rose, Maria Felice Arezzo, Mario Amore, Alessandro Cuomo, Sergio De Filippis, Silvestro La Pia, Marta Pasqualini, Alessandro Pirani, Riccardo Torta, Andrea Fagiolini

**Affiliations:** 1 Department of Methods and Models for Economy, Territory, and Finance, Sapienza University of Rome, Rome, Italy; 2 Department of Neuroscience, Ophthalmology, Genetics and Infant‑Maternal Science, (IRCCS) Ospedale Policlinico San Martino, University of Genoa, Genoa, Italy; 3 Department of Molecular Medicine, University of Siena, Siena, Italy; 4 Neuropsychiatric Clinic, Villa Von Siebenthal, Genzano di Roma, Italy; 5 Dipartimento di Salute Mentale, ASL Napoli 3 Sud, Naples, Italy; 6 Center for Cognitive Disorders and Dementia, Health County of Ferrara, Cento, Italy; 7 Department of Neuroscience, University of Turin, Turin, Italy

**Keywords:** Covid-19, Vulnerability, Mental health, Psychiatry, Public heath response, Italy

## Abstract

COVID-19 pandemic has exacerbated the pre-existing vulnerabilities and inequalities in societies. In this paper we analyse the categories that have suffered more than others from the pandemic and the restrictions on social life in terms of mental health. We rely on the Serendipity project based on a survey administered between November 2021 and February 2022 to a sample of Italian physicians (n = 1281). The survey aimed to assess the perception of general practitioners, paediatricians, geriatricians, and mental health specialists (psychiatrists, neurologists, child neuropsychiatrists), about changes in the mental health of the population as an effect of the COVID-19 pandemic and the lockdown. The strategies implemented by the doctors interviewed in terms of the intensity of the prevention, emergence, and treatment of mental health interventions, and their association with physicians’ characteristics and their opinions on patient vulnerability have been illustrated by means of a multiple correspondence analysis. An overall result of the survey is the consensus of doctors on the worsening of mental health in general population, especially among their patients, due to the pandemic and on the onset of new discomforts. The most exposed individuals to the risk of onset or worsening of mental disorders include women, young people, and patients with psychiatric comorbidity. The paper also illustrates the interventions put in place by the physicians and deemed necessary from a public heath response perspective, that include providing psychoeducation to the general population, improving telehealth services, and increasing financial and human resources for community-based care.

## Introduction

The pandemic is an asymmetric shock: both the health emergency, with its consequences in terms of mortality and morbidity, and the subsequent containment measures, which aimed to contain the spread of the virus and avoid overwhelming the health systems, led to a profound crisis that affected different countries and population groups in different ways (European Commission, 2021). Indeed, the pandemic has exacerbated the pre-existing vulnerabilities and inequalities in societies (Kawachi, 2020). Vulnerability refers to the set of conditions determined by physical, social, economic, and environmental factors or processes, which increase the susceptibility of an individual or a community to certain risks (definition based on the Hyogo Framework for Action 2005–2015 https://www.unisdr.org/2005/wcdr/wcdr-index.htm). This  includes the risk of suffering for the consequences of the epidemic on mental health. Vulnerability can be linked to physical or previous health conditions or social conditions: there may be groups/individuals who have more difficulties in avoiding exposure to the virus, because their working or living conditions do not allow for example to live in healthy, uncrowded places, or to be able to resort to treatment quickly and effectively.

After the first phase of the pandemic, in which it seemed that the virus affected all members of the population in an undifferentiated way, it became clear that the virus acted selectively, choosing the most vulnerable, in terms of age (the oldest population) and social condition (the most marginalized). The institutions immediately paid particular attention to the needs of the weakest groups and some categories of fragile citizens (by age, illness, income) who enjoy specific protection measures. Less attention has been given to the worsening mental health that led to the rise of different vulnerabilities: for example, women working in the health sector; old individuals left alone in retirement homes; young people, confined to their homes/rooms and forced into distance learning and virtual relationships with their peers. The combination of the many adverse conditions in terms of social exclusion and precariousness of general living conditions with the restrictions imposed by the pandemic can have multiplicative effects on mental distress.

In this work, we address the issue of the effect of the pandemic on the mental health of the population as witnessed by the health operators, in particular general practitioners and specialists, who, during the crisis, have taken care of their patients, both those already with psychiatric or neurological problems and those who have experienced for the first time some mental distress. Our analyses are based on the results of the *Serendipity* survey administered between November 2021 and February 2022 to 1281 physicians, operating in Italy. The survey collected the operators’ perception of changes in the mental health of the population that emerged during the Covid-19 pandemic according to their own experience, and how these changes impacted on the normal clinical and professional activities of the doctors themselves. The results, in addition to the clinical focus, allow us to identify the population groups which, according to the physicians’ experience, have suffered more than others from the pandemic and the restrictions on social life. Based on previous empirical studies on the topic, we expect the perception of doctors to show the greater vulnerability of women compared to men, of young and older people compared to adults, of foreign citizens compared to Italians. Furthermore, we expect that doctors have had to implement specific strategies for the care and treatment of mental pathologies in the dramatic pandemic situation.

The paper is structured as follows: after a Background section, that illustrated the context and the empirical studies found in the literature relevant to our work, we describe the *Serendipity* Survey, that is our data source, and test the perceived vulnerabilities among different groups of population; then, the strategies implemented and/or suggested by the doctors interviewed in terms of the intensity of the prevention, emergence, and treatment of mental health interventions and their association with physicians’ characteristics and their opinions on patient vulnerability are illustrated by means of a multiple correspondence analysis (MCA); the Discussion and Conclusions sections summarize our main results and deals with the strengthens and limitations of the *Serendipity* project and our own approach.

## Background

In the months following the start of the first lockdown, many investigations were carried out to monitor how the truly unprecedented situation of confinement and restriction was impacting people's quality of life, including mental health. From a study published in Lancet based on a meta-analysis of the studies conducted around the world and published between January 1, 2020 and January 29, 2021, it emerges a strong association between daily SARS-CoV-2 infection rates and depressive and anxiety disorders which increased by 27.6 and 25.6%, respectively (COVID-19 Mental Disorders Collaborators, 2021).

The appearance of symptoms of psychological distress has often been observed in the event of the spread of infectious diseases. In particular, the most recent epidemics, from SARS in 2003 to H1N1 in 2010, have been accompanied by an increase in anxiety, agitation, depression, or panic attacks (He et al., 2020). In general, the disasters cause much concern among the population, not only related to the fear for own health, but also in connection with the financial and social uncertainty. In the case of COVID-19, the negative effects may have been exacerbated by the severity of virus containment actions, both locally (such as prolonged quarantine) and globally (restricted movement and closing borders). Also, the initial lack of an effective treatment against the virus and confusion about the vaccines could have contributed to the increase of the psychological discomfort, boosted by media overexposure of the people (Chu et al., 2022).

Several studies have reported the early mental health consequences of the COVID-19 pandemic (Singh et al., 2020; Sepulveda et al., 2020), and specifically in Italy (Amendola et al., 2021; Fiorillo et al., 2020).

The research on the topic unanimously confirms that some population categories were affected more than others: females more than males and younger over older age groups, both for major depressive disorder and for anxiety disorders. While from a biological point of view females’ health appear to have been more protected—confirming what is observed during periods characterized by high mortality such as epidemics and famines (Zarulli et al., 2018)—the social consequences were disproportionately attributed to women.

Women have been more exposed to job and economic insecurity than men and faced increased risks of violence and abuse (e.g. OECD, 2020). According to the data of Italian National Institute of Statistics (Istat), Italy has registered a sharp increase in the number of telephone calls to the toll-free helpline: during the lockdown there were 5031 valid calls to 1522, + 73% over the same period in 2019. A total of 2013 victims asked for help (+ 59%).

Looking at the labor market consequences, Istat reports 440,000 fewer employed individuals in December 2020 compared to the previous year, 70.9% of which are women. Moreover, women bear greater burdens than men due to the pandemic: stay-at-home impositions, extended periods of school closure, the unavailability of paid services (such as laundries restaurants, baby-sitters, etc.) as well as the impossibility to benefit from informal care are all factors that have contributed to create a greater demand of unpaid domestic and care work within the households during the pandemic (Rosina et al., 2020; Zannella et al., 2020).

The pandemic had several negative outcomes also for children. Schools closure and social distances from their peers and grandparents resulted in social isolation and emotional distress. Distance learning impacted not only on educational outcomes but also on physical and mental health. A study conducted worldwide on the impact of Covid-19 on the undergraduate and graduate students’ lives has shown that children were facing psychological discomfort (Aristovnik et al., 2020); they felt anxious, frustrated, and bored during the lockdown period (Rosina et al., 2020). Those feelings may have an impact on future intentions and expectations in terms of emancipation from parents, transition to adult life and, therefore, formation of new families (Guetto et al., 2020; Luppi et al., 2020).

For the older population the pandemic highlighted some issues that were already known, but not enough debated: above all, the inequalities, isolation, loneliness and discrimination. Social distancing and/or isolation could have had negative repercussions on the physical and mental health of the elderly and increased the risk of ageism (Previtali et al., 2020).

Migrant groups and minorities differ from general population in terms of opportunity to access knowledge and information about COVID-19 and some did not have the socio-economic or technical means (such as internet access) to care for themselves and their families during the lockdown. A particular concern relates to all those who live in crowded conditions, with difficulty in self-isolating and maintaining social distancing (for example in reception centers for immigrants), or in conditions of poor hygiene and with reduced access to clean water (for example in informal settlements). Among foreigners, delay in the COVID-19 diagnoses has been observed (Fabiani et al., 2021), although no differences as far as the impact of the pandemic on the psychological wellbeing has been documented.

In this paper, we addressed the above topic on the base of an original survey carried on in Italy late 2021/beginning 2022. The *Serendipity survey* differs from all the others because instead of addressing families and individuals directly, it collected the point of view of the physicians, in particular psychiatrics, who told not only their perception of the changes that were taking place, but also the repercussions on their professional activity and which strategies have been implemented.

## The *Serendipity* Survey

Between November 2021 and February 2022, a survey was conducted involving 1281 Italian physicians practicing as psychiatrists, neuropsychiatrists, pediatricians, primary care practitioners, geriatricians and neurologists, equally distributed in the three Italian geographical areas: North, Centre and South. The questionnaire was administered online through Google Form. Respondents participated on a voluntary basis, without any remuneration and anonymously after written consent. The type of investigation did not require any ethics committee approval according to the Italian law.

The questionnaire was developed by the Authors of this paper and consisted of four sections: (1) demographic data, including age, sex, years of experience, and city of residence; (2) opinions about the impact of COVID-19 pandemic on mental health in the general population and own community of patients; (3) description of the strategies that they followed to address the mental health impact of their patients; (4) formulation of scenarios for the future evolution of mental distress due to the pandemic and on the role of targeted interventions. Particular attention was paid, on the one hand, to the assessment of the worsening of pre-existing disorders, on the other, to the onset of new pathological situations. Demographic and practice-related physician data are presented in Table 1.

**Table 1:** Demographic and practice-related physician data (n = 1281)—Italy, 2021–22

Category	Valid % out of responding physicians; Total n = 1281
Males	57.0 (n = 730)
Females	43.0 (n = 551)
Age (M ± SD)	54.04 ± 11.01
Years of experience (M ± SD)	23.70 ± 10.95
Number of patients (M; Range)	626.5 [10–5000]
Psychiatrists	30.2 (n = 387)
Child neuropsychiatrists	5.8 (n = 74)
Neurologists	18.2 (n = 233)
GPs	21.2 (n = 271)
Pediatricians	18.4 (n = 236)
Geriatricians	6.2 (n = 80)

Interviewed doctors (n = 1281) were on average 54.04 years of age (SD = 11.01), most often identified as male (57.0%). The average duration of work experience was 23.70 (SD = 10.95) and the average number of patients was 626.5 patients within a quite large range (min: 10; max: 5000). In order to over-represent the point of view of mental health specialists, the percentage of psychiatrics plus that of neurologists interviewed (n = 694; 54.2%) was higher than that of general practitioners, pediatricians and geriatricians (n = 587; 45.8%).

More than 80% of interviewees observed an increase of mental discomfort after the onset of the COVID-19 pandemic. About 33% of the sample reported an increase of patients with a mental disorder in their community by 1–25%; 34% reported an increase by 26–50%; and 26.9% by 51–75%.

Agitation, anxiety, and mood disorders were the symptoms most often reported by the interviewees, with no important differences between the various medical specialties (Cuomo et al., 2022).

As for the major vulnerabilities found, the physicians interviewed agreed in indicating the women rather than men most exposed to the risk of psychological distress due to Covid-19 and the restrictions imposed by the government, as well as the young individuals with respect to the older ones (Table 2, column a); contrary to our expectation, the great majority of the physicians observed a highest prevalence of mental distress among the natives than among the foreign population.

**Table 2:** Most vulnerable categories according to physicians' experience of Covid-19 impact on mental health (n = 1281)—Italy, 2021–22

Category	Valid percentage out of responding physicians; Total n = 1281 (a)	χ2 test for the association with medical specialization *p* value (df) (b)
Males	36%	0.6156 (5)
Females	64%	
Children (age 0–17)	19%	0.0000 (15)
Young (age 18–29)	40%	
Adult (age 30–64)	34%	
Older people (age 65+)	7%	
Italians	84%	0.2723 (5)
Immigrants	16%	
Psychiatric comorbidity	75%	0.0048 (5)
Non-psychiatric comorbidity	67%	0.2487 (5)

From our findings it also emerges that patients with previous mental disorders were most exposed to negative psychological distress (75% of the physicians agreed on that). If higher levels of anxiety and agitation were noted among the general population, these symptoms worsened more in patients with a previous history of psychological burdens. Patients with a history of trauma suffer from the long-term effect of trauma itself, with symptomatology that could flare up or re-emerge during a situation of psychological uncertainty. Also, 67% of the interviewed doctors observed an increase of mental distress among people with serious health related problems other than psychiatric, namely neurological, cardiovascular, or respiratory pathologies.

The answers are quite homogenous with respect to the medical specialization of the operators (Table 2, column b), except for the age group of the most vulnerable patients, since the opinions differ significantly between the categories of doctors interviewed (χ2 *p* value < 0.000). Also, the impact of the psychiatric comorbidity is differently evaluated among the various specialists in the sample (χ2 *p* value < 0.005).

**Fig. 1:**
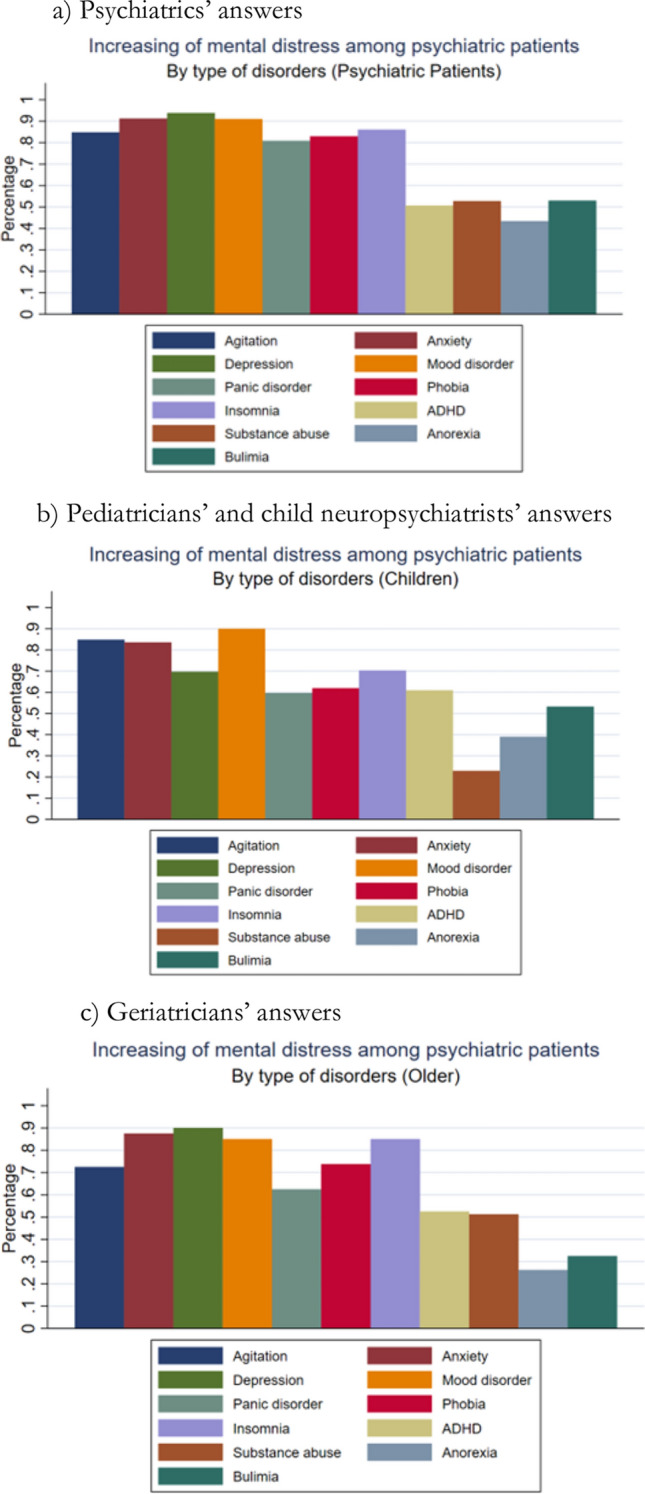
Increase in mental distress by symptoms in patients with psychiatric comorbidity and different specialists, Italy 2021–22. Due to the COVID-19 pandemic, did you observe an increase in the prevalence of: Panel **a** Psychiatrics’ answers. Panel **b** Pediatricians’ and child neuropsychiatrists’ answers. Panel **c** Geriatricians’ answers

The different opinions that we have found about the vulnerability factors are clearly practice-related because the type of patient varies according to the different medical disciplines involved and consequently also the severity and type of symptoms observed. In fact, if we analyze the distributions of symptoms that doctors consider to be the most worsened among patients with previous mental health problems, distinguishing between geriatricians, pediatricians/child neuropsychiatrists, and psychiatrists, we obtain different images (Fig. 1). Those who mainly deal with the elderly have observed above all an increase of the anxiety and depression among patients with existing psychiatrics symptoms while those who take care of children and young people health testified above all to an increase in mood disorders due to the pandemic situation.

Of particular interest are the strategies implemented by the doctors interviewed in terms of the intensity of the prevention, emergence, and treatment of mental health interventions (Kichloo et al., 2020; Farsalinos et al., 2021; ISS, 2020). The survey collected several data on the topic, asking doctors what strategies they have adopted in their practice and to follow up their patients, especially the psychiatric ones, during the most difficult phases of the pandemic. In addition, doctors were asked for the opinion of the most important interventions that can or should be made to better address the post-pandemic psychological distress situation in the future. Again, we expect that the responses of physicians vary according to their experience and specialization, as well as it according to the different vulnerabilities of patients which require different interventions. To have a complete picture of the answers of the interviewees in terms of interventions adopted and suggested, that considers the aforementioned complex associations, we performed a multiple correspondence analysis (MCA) that is going to be illustrated in the next section.

## Implemented Strategies and Suggested Interventions: A Multiple Correspondence Analysis

### Method

For this study, we used multiple correspondence analysis (MCA), an inductive statistical technique useful for exploring relations among multiple categorical variables but not for hypothesis testing (Clausen, 1998). MCA allows researchers to reduce a complex data matrix into a simpler one without losing meaningful information and allows the understanding of the complex relationships among the categorical variables of interest. Under a mathematical point of view, MCA is about the search of eigenvalues and eigenvector of the so-called Burt matrix, the matrix of all two-way cross tabulations of the categorical variables in the analysis.

A powerful tool in the interpretation is the graphical representation of the relationship among the variables in a subspace of low dimension that preserves as much as possible the original complex framework of links among categories. There are three important elements in the correspondence analysis: (1) profiles, (2) masses, and (3) chi-square distances (Greenacre, 1994).

The profiles are simply relative frequencies with respect to row or columns marginal frequencies; in other words, in order to compute row/column profiles the frequencies inside the contingency table are divided by the row/column marginal frequencies. Each profile represents a point in space, which is projected in the subspaces identified as a linear combination of the original ones by mean of the eigenvectors of the Burt matrix.

The profiles are of two types: *active* if the variable contributes to the determination of eigenvalues and eigenvectors and *illustrative* if it does not.

In MCA categories of variables with similar profiles are placed as points clustered together in space; on the other hand, categories with dissimilar profiles are displayed as points that are far away from each other.

Masses are obtained by dividing each row/column marginal frequencies by the sample size and they give a measure of the importance of each category.

Distances between profiles are computed as chi-square distances, which are weighted Euclidean distances with weights that are the inverse of its relative frequency. The use of a chi-square distance is motivated by the preservation of the importance of very small relative frequencies.

In this study, we used multiple correspondence analysis to explore (1) the relation among doctors’ characteristics and the strategies used to treat patients with psychological issues during the pandemic and (2) the relation among physicians’ characteristics and the most important interventions that can be made to address / improve the post-pandemic psychological distress situation.

Multiple Correspondence Analysis allowed us to examine the associations among the variables of interest visually, concluding that categories closed in space are associated and are responsible for the overall pattern of dependency.

When there are multiple profiles and it is not possible to massively simplify the data matrix, it is very useful to use cluster analysis together with MCA.

Unlike MCA, cluster analysis aims primarily to provide homogeneous groupings of subjects and/or variables based on a multivariate similarity metric. When applied in a complementary fashion, MCA and cluster can be used to enhance each other’s interpretation of results (Gorman & Primavera, 1983).

### Active Variables

The categorical variables that describe the doctors’ characteristics are: (1) Specialization with levels Child Neuropsychiatrists (coded as Child_Neuro), General pediatricians (Gen_Pediat), General Practitioners (Gen_Pract), Geriatrician, Neurologists (Neuro), Psychiatrists (Psych); (2)Years of experience with levels <  = 15yrs, 16–25yrs, 26-35yrs, 36 + ; (3) Number of patients with levels <  = 300, 301–700, 700 + ; (4) Geographical area where they doctors practice with levels Center, North, South.

The main interest variables are (1) *During the pandemic, what assistance strategies did you use to treat your patients with mental health problems?* with levels Remote counselling and/or remote psychotherapy (Remote_psych/couns), As before, but with personal safe devices (Same_PSD), Increase of contacts/availability via telephone and email (Tel&Mail), Other; (2) *In your opinion, what are the most important interventions that can be made to address / improve the post-pandemic psychological distress situation?* with levels Educational interventions on mental illness aimed at the general population (Edu_Ment_Ill), Strengthening of mental health services (MentHealth_Str), Increase of resources for outpatient/territorial activities (Resour_Str), Strengthening of telemedicine services or Development of automated systems for computer-based self-therapy (Telemed_Str), Other.

### Illustrative Variables

This set comprises two variables: (1*) Did the presence of psychiatric comorbidities accentuate the appearance of psychic manifestations (disorders) in your patients?* with levels yes and no; (2) *In your experience, with respect to the age range, which of the following subjects were at greater risk?* with levels kids & teens (< 18 years old), young (18–30 years old), adults (31–65 years old), older adults (66 or older).

## Results

### MCA on Doctors’ Characteristics and the Assistance Strategies Used to Treat Patients with Mental Health Problems

The results of the MCA displayed 15 eigenvalues, indicating that 15 independent dimensions had been identified. To raise the interpretability of the results, we decided to retain the first six dimensions, which explained approximately half of the total inertia. Table 3 contains the projected profile coordinates on the first three dimensions, along with the v-test that identifies those categories that are significantly contributing to the inertia of the dimension. Categories with a v-test value greater than 2.0 (negative or positive) are significant.

**Table 3:** Coordinates of the categories of active variables in the subspace of the first three dimensions—variables included: doctors’ characteristics and *the* assistance strategies used to treat patients with mental health problems

		Coordinates		
		Dim.1	Dim.2	Dim.3
Specialization	Child_Neuro	*0.052*	− 1.038	− 0.323
	Gen_Pediat	0.318	0.450	0.853
	Gen_Pract	− 0.837	0.811	− 0.239
	Geriatrician	0.269	− 0.713	0.948
	Neuro	0.366	− 0.232	0.372
	Psych	0.107	− 0.356	− 0.711
Years of experience	< = 15yrs	1.094	0.495	0.132
	16-25yrs	0.124	− 0.983	− 0.883
	26-35yrs	− 0.403	− 0.251	0.640
	36 +	− 1.158	1.146	− 0.749
No. of patients	< = 300	1.291	0.768	− 0.204
	301–700	0.100	− 0.667	0.139
	700 +	− 0.930	0.343	− *0.044*
Geographical area	Center	− 0.158	− 0.332	0.240
	North	− 0.325	− 0.120	0.078
	South	0.402	0.356	− 0.250
Interventions to address distress	Remote_psych/couns	− *0.002*	− 0.478	− 0.318
	Same_PSD	− 0.155	− 0.145	− 0.168
	Tel&Mail	0.287	0.347	− 0.362
	Other	− 0.151	0.264	1.129
	Eigenvalue	0.318	0.267	0.232
	Explained inertia	10.584	8.916	7.745

In italics the categories with non-significant v test

As we said before, profiles that are close together in space are responsible for the dependency pattern that we observe in the data, and therefore a crucial part of MCA is the understanding of the profile closeness. When the relevant dimensions are more than two, as in our case, and a visual inspection is not feasible, a very effective way is to find the clusters of similar (i.e. close together) categories. We used the k-means algorithm (Forgy, 1965) to accomplish the task and tested the robustness of our results using hierarchical (Ward, 1963) and fuzzy clustering (Bezdek, 1981) approaches. Apart from slight variations, clusters composition was quite stable over the methods giving strength to our conclusions.

The clusters emerged were the following three:

- Cluster 1: Tel&Mail, Gen_Pediat, Geriatrician, < = 15yrs, < = 300, South, AR_kids & teens, PPC_No;
- Cluster 2: Remote_psych/couns, Child_Neuro, Neuro, Psych, 16–25 years, 301–700, AR_young;
- Cluster 3: Same_PSD, Other, Gen_Pract, 26-35yrs, 36 +, 700 +, Center, North, AR_adults, AR_older adults, PPC_Yes

It emerges a clear pattern of different strategies undertaken according to the doctors’ characteristics: General pediatricians and geriatricians with no more than 15 years of experience and with 300 patients or less in the south of Italy, whose patients are without psychiatric comorbidity, have adopted a strategy based on the intensification of email and telephone contacts.

On the other side, neurologists, child neurologists and psychiatrists with an experience between 15 and 25 years and a number of patients between 301 and 700 opted for remote counseling. This type of doctors believed that the young people were those exposed to higher risk.

Finally, general practitioners with vast experience (at least 26 years) and a great number of patients (700 or more) located in the north or the center part of Italy who believed that adults and older adults with previous mental disorders were the categories at higher risk, opted for maintaining visits and contacts with patients protected with personal and environmental equipment or adopted other strategies.

### MCA on Doctors’ Characteristics and the Most Important Interventions that can be Made to Address/Improve the Post-pandemic Psychological Distress Situation

For this analysis we implemented the same methodology as before, that is we first performed MCA on all categorical variables, then we retained few dimensions that explained a fair amount of total inertia and finally we clustered up similar profiles.

The MCA displayed 16 dimensions, and we retained the first 7 that explained approximately 52% of the total inertia. Table 4 shows the projected profile coordinates on the first three dimensions, along with the v-tests.

**Table 4:** Coordinates of the categories of active variables in the subspace of the first three dimensions—variables included: doctors’ characteristics and the most important interventions that can be made to address/improve the post-pandemic psychological distress situation

		Coordinates		
		Dim.1	Dim.2	Dim.3
Specialization	Child_Neuro	*0.104*	− 0.923	0.599
	Gen_Pediat	0.301	0.456	1.273
	Gen_Pract	− 0.878	0.836	− 0.418
	Geriatrician	0.326	− 0.925	− 0.514
	Neuro	0.397	− 0.203	0.145
	Psych	0.105	− 0.374	− 0.579
Years of experience	< = 15yrs	1.112	0.534	− 0.263
	16-25yrs	0.129	− 0.946	− 0.563
	26-35yrs	− 0.399	− 0.271	0.743
	36 +	− 1.208	1.076	− 0.735
No. of patients	< = 300	1.254	0.838	− 0.225
	301–700	0.122	− 0.643	0.143
	700 +	− 0.934	0.270	− 0.036
Geographical area	Center	− 0.131	− 0.344	− 0.243
	North	− 0.312	− 0.166	0.108
	South	0.369	0.405	0.090
Assistance strategies	Edu_Ment_Ill	− 0.108	− 0.159	0.095
	MentHealth_Str	0.103	− 0.131	− 0.546
	Other	− *0.260*	0.593	− *0.320*
	Resour_Str	− *0.068*	0.657	0.711
	Telemed_Str	*0.105*	*0.077*	0.762
	Eigenvalue	0.316	0.266	0.230
	Explained inertia	9.87	8.306	7.179

In italics the categories with non-significant v test

The following two clusters emerged:

- Cluster 1: MentHealth_Str, Geriatrician, Neuro, < = 15yrs, < = 300, Center, AR_older adults;
- Cluster 2: Edu_Ment_Ill, Other, Resour_Str, Telemed_Str, Child_Neuro, Gen_Pediat, Gen_Pract, Psych, 16-25yrs, 26-35yrs, 36 +, 301–700, 700 +, North, South, AR_adults, AR_kids & teens, AR_young PPC_No, PPC_Yes

The pattern in the data is once again clear, and it shows that geriatricians and neurologists in the center of Italy with 15 years of experience or less who have 300 or less patients believe that the most important intervention would be the strengthening of mental health services.

The other typologies of doctors represented in cluster 2, proposed a more articulated form of interventions spanning from educational interventions on mental illness aimed at the general population, to the increase of resources and the reinforcement of telemedicine.

## Discussion

Since its emergence in December 2019, COVID-19 has had enormous social, behavioral and economic consequences around the globe. The effects of the COVID-19 pandemic go far beyond physical health, impacting individuals’ and communities’ everyday lives and well-being, including in the domains of mental health. However, the pandemic has not disrupted everybody’s lives in the same way.

Vulnerable and marginalized groups are disproportionately exposed to negative impact (Kawachi, 2020). Namely, vulnerable people are those at higher risk of poor physical, mental, and social health (Aday, 2002). The pandemic effects have been different between population groups not just in terms of COVID-19 morbidity and mortality rates, but also in terms of the consequences of the implemented restrictions and emergency lockdown measures (Bambra et al., 2020). Clinically vulnerable groups in the COVID-19 pandemic include the older people (Gardner et al., 2020; Sadruddin & Inhorn, 2020) and people with chronic diseases or other underlying risk factors (Gao et al., 2021).

In the case of mental health, vulnerable groups are not necessarily the same groups as those who face greater clinical risk of COVID-19. With this study, we showed that the most exposed individuals to the risk of onset or worsening of mental disorders include young people more than the older one and the woman more than men. We also found that patients with previous mental distress were particularly affected by the symptoms worsening due to COVID-19 pandemic. If an increase in anxiety and agitation level was noted among the general population, it was even more severe in patients with a previous history of psychiatric disease.

Many adverse conditions in terms of social exclusion and precariousness of general living conditions with the restrictions imposed by the pandemic can have multiplicative effects on mental distress. Other elements may have played a role, for example the lack of clarity—at least in the early stages of the pandemic—on possible treatments and vaccines, also fueled by people's excessive exposure to unscientific information, if not disinformation and false reports (Germani & Biller-Andorno, 2021). On this last topic, since the first vaccine campaign was promoted at the beginning of 2021, the Italian population has been dealt with a series of fake news about COVID-19 vaccines and their possible adverse effects: erroneous information about a high risk of mortality after vaccine injection or use of dangerous substances in vaccine formulations have prevented many Italians from vaccination, generating more increased agitation and anxiety levels. In this context, social media played a crucial role in increasing psychological distress: from our findings, the most exposed mediatic population, like women and young adults, resulted more subjected to psychological burdens, as well as the most fragile patients as to mental health who may have distorted the information received and encountered a deterioration of their conditions.

On this perspective, the impact of COVID-19 on mental health represented a big challenge for mental health providers. Indeed, while the Italian public response was substantial in the early stages of the spread of the virus as far as its containment and the care of infected people was concerned (Torri et al., 2020), its attention toward mental health consequences was less and delayed. However, from our sample of doctors, both general practitioners and specialized in the treatment of neurological and psychiatric disorders, it emerges that the response by individual operators to their patients, especially if with comorbidities, was decisive and consistent with the professional role played and with the different vulnerabilities shown by their patients. They also offered a rich range of actions and proposals for the future from which specific indications of interest for the policies emerge: the specialized respondents mainly recommended providing psychoeducation to the general population for early detection of mental illness and developing strategies to reduce the impact of COVID-19-related stress and improving telehealth services; the need to increase financial and human resources for community-based care clearly emerges from general practitioners. There is a consensus that providing and improving the availability of telemedicine services could reduce the impact of future challenges related to the pandemic.

Our study is not without limitations (Cuomo et al., 2022). First, the survey was conducted on medical doctors and not on patients, so no conclusions for the impact of COVID-19 on mental health in an epidemiological perspective are possible. Second, this study is based on the physicians’ opinions and perceptions, thus it appears as a qualitative study, with an emphasis on subjective experiences more than objective and measured ones. With these data it is not possible to properly evaluate the impact of the pandemic on mental health, which can only be allowed by a survey design that provides for the collection of pre-during-and post-pandemic information. Instead, we interviewed doctors only once—between November 2021 and February 2022—and asked them to make a personal assessment of the effect of Covid.19 on the mental health of their patients. Third, our sample was not representative of the whole population of doctors who practice in Italy and, moreover, we oversampled psychiatrics and neurologists. Finally, given that participants were recruited through e-mails and social media, and their answering was voluntary, we cannot exclude a sampling bias. Furthermore, the physicians’ opinions could have been affected by their own mental distress due to the pandemic situation and by the level of physician burnout, which we did not control for.

Despite these limitations, the *Serendipity* study provides the point of view of doctors of different specializations and has offered an original contribution to the issue of the impact of COVID-19 on psychological wellbeing and has allowed us to find non-trivial results relating to the various vulnerabilities to mental distress. Moreover, the variety of indications provided by doctors personally facing the pandemic emergency, that has deeply challenged the organization of the national health system, suggests that many actions should be taken not only to prepare for future similar shocks, but also to reorganize from now the mental health services. This is useful for the prevention of increased psychological distress and for planning psychological interventions to improve mental health resilience during public health emergencies.

## Conclusions

The overall result of the original *Serendipity* survey administrated to 1281 medical doctors is the consensus of the Italian physicians, both general practitioners and specialists, on the worsening of mental health in general population due to the pandemic. Agitation, anxiety, and mood disorders and the most observed symptoms, especially among the patients with a history of psychiatric and/or neurological disease: for them the pandemic situation heavily worsened the mental status. As far as the different vulnerabilities are concerned, our hypotheses are only partially confirmed by the results. Indeed, the doctors testified to a greater fragility of women than men and the worrying emergence of mental discomfort in young people more than in the elderly, while they did not suggest a greater vulnerability of non-Italian citizens.

The heterogeneity of the answers mirrors that of the sample interviewed, which includes various medical specializations, each with its own sensitivity. To account for this complexity, we applied a multiple correspondence analysis. It emerges a clear pattern of different strategies undertaken according to the doctors’ characteristics, namely their specialization, the length of their experience, the geographical context. Also, the suggested interventions that can be made to address/improve the post-pandemic psychological distress situation are specialist-based and depend on doctor’s characteristics, spanning from strengthening of mental health services—mainly suggested by neurologists and geriatricians—to educational interventions on mental illness aimed at the general population, to the increase of resources and the reinforcement of telemedicine.

## Data Availability

The datasets used and/or analyzed during the current study are available from the corresponding author upon request.

## Appendix

See Tables 3 and 4.
